# Pediatric chest radiograph interpretation in a real-life setting

**DOI:** 10.1007/s00431-024-05717-x

**Published:** 2024-08-12

**Authors:** Bar Rotem-Grunbaum, Oded Scheuerman, Oren Tamary, Yaniv Lakovsky, Vered Shkalim Zemer, Lotem Goldberg, Niv Soffair, Yarden Bulkowstein, Shahar Hendelsman, Gil Amarilyo, Noga Yaniv, Yoel Levinsky

**Affiliations:** 1 Department of Pediatrics B, Schneider Children’s Medical Center of Israel, Petah Tiqva, Israel; 2 Faculty of Medicine, Tel Aviv University, Tel Aviv, Israel; 3 Pediatric Emergency Department, Schneider Children’s Medical Center of Israel, Petah Tiqva, Israel; 4 Pediatric Radiology Department, Schneider Children’s Medical Center of Israel, Petah Tiqva, Israel; 5 Clalit Health Services, Petach Tikva, Israel; 6 Pediatric Rheumatology Unit, Schneider Children’s Medical Center of Israel, Petach Tikva, Israel

**Keywords:** Chest radiographs, Emergency medicine, Pulmonology, Pneumonia, Radiography

## Abstract

Chest radiography is a frequently used imaging modality in children. However, only fair to moderate inter-observer agreement has been reported between chest radiograph interpreters. Most studies were not performed in real-world clinical settings. Our aims were to examine the agreement between emergency department pediatricians and board-certified radiologists in a pediatric real-life setting and to identify clinical risk factors for the discrepancies. Included were children aged 3 months to 18 years who underwent chest radiography in the emergency department not during the regular hours of radiologist interpretation. Every case was reviewed by an expert panel. Inter-observer agreement between emergency department pediatricians and board-certified radiologists was assessed by Cohen’s kappa; risk factors for disagreement were analyzed. Among 1373 cases, the level of agreement between emergency department pediatricians and board-certified radiologists was “moderate” (*k* = 0.505). For radiographs performed after midnight, agreement was only “fair” (*k* = 0.391). The expert panel identified clinically relevant disagreements in 260 (18.9%) of the radiographs. Over-treatment of antibiotics was identified in 121 (8.9%) of the cases and under-treatment in 79 (5.8%). In a multivariable logistic regression, the following parameters were found to be significantly associated with disagreements: neurological background (*p* = 0.046), fever (*p* = 0.001), dyspnea (*p* = 0.014), and radiographs performed after midnight (*p* = 0.007).

*Conclusions*: Moderate agreement was found between emergency department pediatricians and board-certified radiologists in interpreting chest radiographs. Neurological background, fever, dyspnea, and radiographs performed after midnight were identified as risk factors for disagreement. Implementing these findings could facilitate the use of radiologist expertise, save time and resources, and potentially improve patient care.

**Table:** 

**What is Known:** *• Only fair to moderate inter-observer agreement has been reported between chest radiograph interpreters.* *• Most studies were not performed in real-world clinical settings. Clinical risk factors for disagreements have not been reported.*
**What is New:** *• In this study, which included 1373 cases at the emergency department, the level of agreement between interpreters was only “moderate.”* *• The major clinical parameters associated with interpretation discrepancies were neurological background, fever, dyspnea, and interpretations conducted during the night shift.*

**Supplementary Information:**

The online version contains supplementary material available at 10.1007/s00431-024-05717-x.

## Introduction

Chest radiography is one of the most frequently used imaging modalities in children, [1, 2] and aids in diagnosing several serious pediatric conditions, including pneumonia and its complications, pneumothorax, and congenital heart malformations [3]. The most frequent use of chest radiographs (CR) is as a first-line imaging modality for diagnosing community-acquired pneumonia, although clinical guidelines do not routinely recommend this in uncomplicated circumstances [4]. While their use has decreased over the past decade, CRs are still frequently performed. A prevalence of 80% was reported in emergency departments (ED) in the United States for children diagnosed with community-acquired pneumonia [2].

Misinterpretation of CR can lead to over-diagnosis and excessive treatment, or to under-diagnosis and potentially harmful consequences [5]. Despite its availability and common use, CRs are considered relatively difficult to interpret [6]. Some studied have reported only fair to moderate levels of inter-observer agreement, even among board-certified pediatric radiologists [7, 8], although their inter-observer agreement is likely better than that of other expertise [8–10]. Trainees, as pediatric residents and fellows, have shown lower levels of inter-observer agreement than board-certified physicians when each group was studied separately. [9, 11] Notably, radiologist interpretation was considered a “gold standard” in some previous studies [5, 11, 12].

For the reasons described, it is customary in many medical institutions worldwide to provide interpretation by a board-certified radiologist in addition to the interpretation by the ED pediatricians. Interpretations by a radiologist have added value for patient safety and quality assurance [5]. Nevertheless, the burden of advanced modern imaging modalities has significantly grown, placing substantial workload on radiologists [13]. Identifying the circumstances in which a radiologist’s interpretation is most needed could facilitate effective management of medical resources. Furthermore, this approach could guide pediatric providers in determining when they should seek a radiologist’s interpretation of imaging results.

A major limitation of relevant published studies is that they were mostly carried out in controlled, non-clinical settings and involved reviewing image sets not within a real-world clinical context [7–11]. In these settings, it is difficult to estimate the consequences of misinterpretation and the effect of the clinical settings on interpretation.

We aimed to examine the level of agreement between ED pediatricians and radiologists in interpreting CR of pediatric patients presenting to the ED. In this real-life setting, we hypothesized that the level of agreement would be higher than previously reported, as the patients’ clinical conditions could provide useful clues to interpret the CRs even for less experienced physicians. In addition, we aimed to estimate the extent of over/under treatments related to interpretation discrepancies; and to identify risk factors, particularly clinical parameters, for these discrepancies. Identifying these risk factors could potentially facilitate prioritizing CRs for a radiologist’s interpretation.

## Material and methods

### Patients and settings

This cross-sectional study was conducted in a tertiary pediatric hospital. Included were patients aged 3 months to 18 years, who were admitted to the ED during the year 2019. This year was chosen to avoid possible biases related to the particular conditions of the COVID-19 pandemic, starting in Israel in February 2020. Inclusion criteria were the conduct of a CR during the patient’s visit, not during the regular hours of radiologist interpretation (as explained below).

- “Radiologist interpretation”: In our hospital, during working hours, a board-certified radiologist is present at the hospital and provides an “on-line” interpretation of the CR. This interpretation of imaging is published during the radiologists’ working hours and almost never outside these hours, i.e., night shifts and weekends (a full timetable is provided in supplementary Fig. 1).
- “ED pediatricians”: Regularly, board-certified pediatric ED specialists, board-certified pediatricians, ED fellows, and pediatric residents are present in our ED until 00:00, after which only pediatric residents are present.

**Fig. 1:**
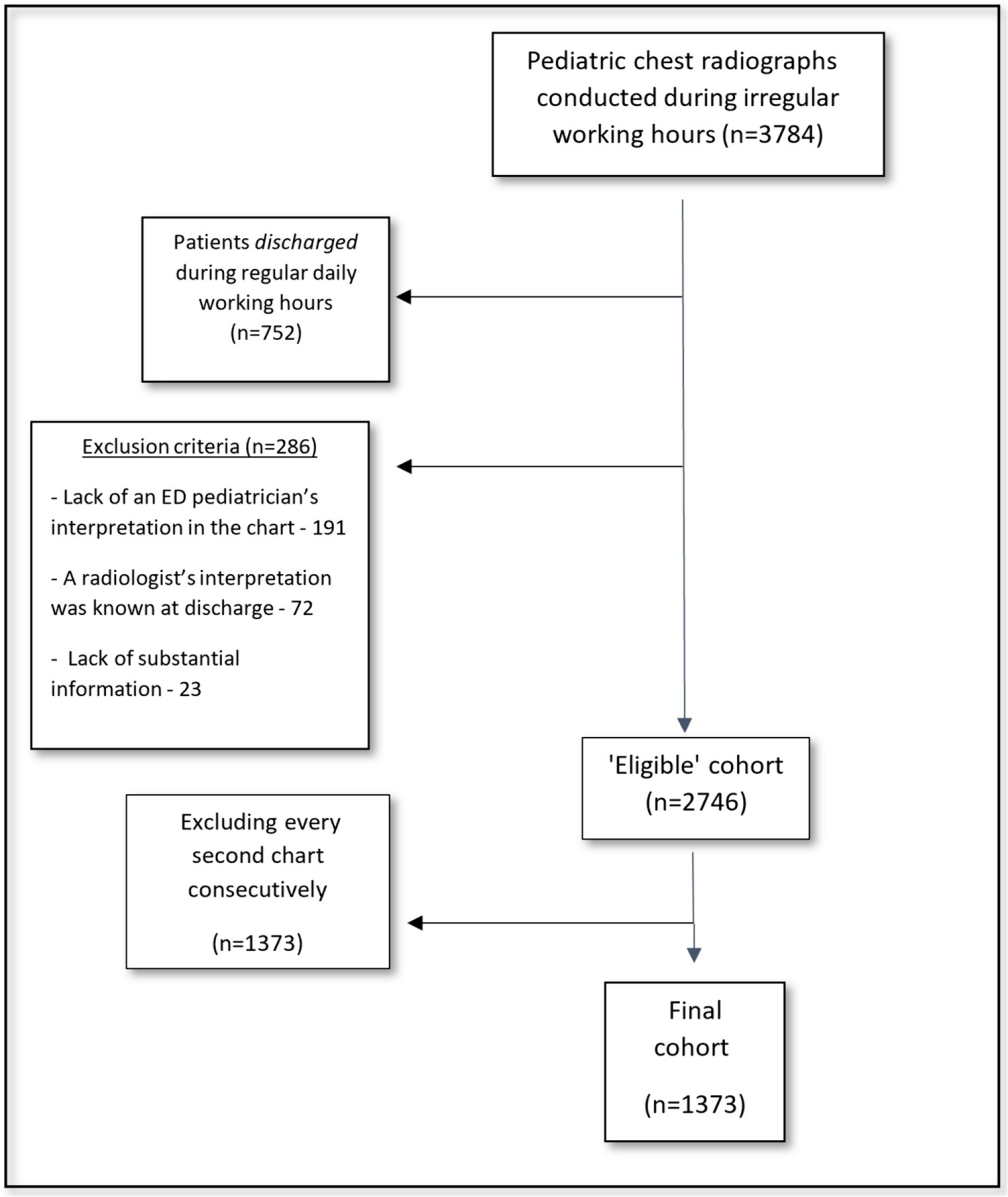
Study flowchart

To ensure that no radiologist diagnosis influenced the interpretation by the ED pediatricians, we included only CRs that were performed during night shift hours or during the weekend. The time of discharge was also considered. Specifically, patients who were discharged at times when a radiologist’s interpretation could have been available were not included, to avoid skewing the results. Excluded were CRs that were performed due to trauma, when a radiologist’s interpretation was not available, or when the interpretation by the ED pediatrician was not recorded in the medical chart. Further, CRs were excluded if the review of the medical records raised suspicion that the ED pediatrician may have received a radiologist's interpretation before discharging the patient (i.e., either an explicit statement that the interpretation was by a radiologist; or alternatively, the wording of the chart interpretation was similar to that of radiologists).

The study was approved by the Research Ethics Board of Rabin Medical Center (Approval No. RMC-21–0293).

### Study design

In the first step, all the included CRs were retrieved through the computerized system. The system was searched for all the consecutive chest radiographs in the relevant period. Each CR was retrieved together with the patient's medical chart. Every medical chart was reviewed by a pediatric resident (*BRG*,* SH*, and* YB*) who applied the exclusion criteria when relevant. Demographic and clinical data were collected, in addition to the interpretations of the CRs by the ED pediatricians in the exact wording of their documentation in the medical chart. The radiologists’ interpretations, as were published later, were collected as well. All the relevant data were entered into an Excel file. To reach the predetermined sample size, every consecutive second chart was selected for inclusion in the study, effectively including half of the total CRs.

In the next step, every case was reviewed by a panel of three board-certified pediatricians [*YL*,* LG*, and* VSZ*]. Each member of this “expert panel” had more than 5 years of board-certified experience, and none of them worked in the ED during the study period. Together, they assessed the agreement between the interpretation of the ED pediatricians and the interpretation that was later recorded by the board-certified radiologist. The panel members were instructed to evaluate the cases according to the WHO assessment method of CR (see below) which were previously presented to them. Only disagreements that were considered to have clinical implications (i.e., any management changes, including the initiation or discontinuation of antibiotics, a pulmonologist consultation, referrals for further imaging, etc.) were considered as discrepancies. The experts categorized each case as either “clinically relevant agreement” or “disagreement.” In cases of differing opinions among the panel members, a majority was required. In a limited number of cases, the experts unanimously agreed that the cases could not be attributed to either of the two groups (i.e., “partial agreement”). These cases were excluded from the risk factors analysis (*secondary outcome, below*). The expert panel reviewed the final diagnoses of all the cases as well, and determined for each, the main indication for the CR and the primary diagnosis at discharge.

### Definitions and laboratory methods

Study outcomes and gold standards are as follows:

1. The main outcome of the study was the level of agreement between ED pediatricians and radiologists, estimated by Cohen’s kappa [i.e., without the need to define a “gold standard”].[14]
2. Secondary outcome: clinical risk factors for disagreements. Those were calculated without predetermining any gold standard as well;
3. Secondary outcome: over-treatment versus under-treatment. For this outcome, the gold standard for chest radiograph interpretation was considered as the interpretation by the radiologists (see “Limitations”, below).

We defined CR interpretation according to the WHO assessment method of CR for the diagnosis of pneumonia in children, as 0—“no consolidation/infiltrate/effusion”; 1—“other (non-end-point) infiltrate”; 2—“significant pathology end-point consolidation”; and 3—“pneumonia with pleural effusion” [15]. The conclusions of radiographs interpretations were determined according to the WHO definitions as “pneumonia with pleural effusion,” “primary end-point pneumonia,” “other infiltrate,” and “no consolidation/infiltrate/effusion” [15]. Additional diagnoses that were coded included suspected foreign body aspiration, pneumothorax, and other (Table 1).

**Table 1:** Basic demographic and clinical parameters of the patients (*n* = 1373), including chest radiograph features

Age (years); median (IQR)	2.1 [1.1–5.1]
Gender, female	572 (41.7)
Medical background, any	379 (27.6)
Season, October–March	903 (65.8)
Ambulance arrival at the emergency department	108 (7.9)
Antibiotic therapy recommended at discharge^a^	618 (45.0)
Hospital admission	289 (21.0)
Fluid therapy	257 (18.7)
*Level of experience of ED pediatricians* Residents Board-certified pediatricians or ED pediatrician specialists	1068 (77.8) 305 (22.2)
*Main indications for chest radiography* Respiratory symptoms Investigation of fever (without any respiratory signs) Suspected foreign body Chest pain/pressure Other	650 (47.3) 544 (39.6) 54 (3.9) 67 (4.9) 52 (3.8)
*Final diagnoses of the patients at discharge (ED pediatricians)*^*b*^ Respiratory infection, viral Viral infection without overt respiratory involvement Bacterial pneumonia Other bacterial infection Other diagnoses/uncertain	506 (36.9) 184 (13.4) 409 (29.8) 80 (5.8) 194 (14.1)
*Conclusion of chest radiographs (radiologist interpretation)* Pneumonia with pleural effusion Primary end-point pneumonia Other infiltrate No consolidation/infiltrate/effusion Pneumothorax Consistent with foreign body in airways Chest congestion Other	35 (2.5) 251 (18.3) 148 (10.8) 901 (65.6) 7 (0.5) 14 (1.0) 9 (0.7) 8 (0.6)

The data are presented as *n* (%) unless stated otherwise

*IQR* interquartile range, *ED* emergency department

^a^For various indications

^b^The main final diagnosis after review by the expert panel

### Statistical analysis

The sample size was calculated assuming about 25% bacterial pneumonia among the expected cohort [16], with an expected kappa of 0.5, precision rate of 0.06, and a drop rate of about 20%, yielding *n* = 1335 [17]. Continuous variables were calculated as means and standard deviations, and discrete variables as numbers and percentages. Cohen’s kappa was calculated for the whole cohort and among various subpopulations. Kappa results were interpreted as follows: values ≤ 0 as indicating no agreement; 0.01–0.20 as none to slight agreement; 0.21–0.40 as fair agreement; 0.41– 0.60 as moderate agreement; 0.61–0.80 as substantial agreement; and 0.81–1.00 as almost perfect agreement.^14^ Next, we compared, using appropriate statistical analyses, the characteristics of the children, between those who did and did not have discrepancies, between the CR interpretations of the ED pediatricians and of the board-certified radiologists. For these analyses, CRs with partial agreement were omitted. Nominal variables were compared using Pearson’s *χ*^2^ test; continuous variables that matched parametric criteria were compared using Student’s *t*-test; and ordinal variables or continuous variables that did not match parametric criteria were compared using the Mann–Whitney *U* test. Data for some parameters were missing in a minority of patients. No significant differences were observed in missing data between those with and without agreement. Therefore, missing data were omitted from the analysis. A binary logistic regression was performed including age and sex, of parameters reaching *p* < 0.07 (i.e., trend to significance), or other clinically relevant parameters. A *p* value of ≤ 0.05 was considered significant. Data were analyzed using Statistical Package for the Social Sciences (SPSS) statistical software, version 24 (SPSS Inc, Chicago, Illinois).

## Results

### Study cohort

A flowchart of the study cohort is depicted in Fig. 1. A total of 1373 CRs were included in the final cohort. Their characteristics are detailed in Table 1, together with indications for the imaging, primary radiologists’ interpretations, and final diagnoses at discharge.

### CR interpretations and inter-observer agreement

Cohen’s kappa for inter-observer agreement was calculated in various scenarios (Table 2). For the whole cohort, the kappa for agreement between radiologists and ED pediatricians was moderate, 0.505 (95% CI 0.455–0.554). Next, we divided the categories for CR interpretation into two major groups, according to the need for antibiotic treatment. Recalculating Cohen’s kappa accordingly, we found moderate agreement, 0.508 (95% CI 0.459–0.557). The kappa was 0.495 [95% CI 0.436–0.553] for the 1068 CRs interpreted by residents, and 0.512 [95% CI 0.405–0.618] for the 305 CRs interpreted by board-certified pediatricians. Kappa was also calculated according to indication, considering only radiographs that were obtained due to respiratory symptoms or fever, to rule out bacterial pneumonia; Among those, kappa was moderate as well, 0.440 [95% CI 0.411–0.470]. Kappa for other indications was not calculated due to the low number of cases. Finally, kappa calculated for all the radiographs obtained after midnight (*n* = 332) was found to be “fair” 0.391 [95% CI 0.282–0.500].

**Table 2:** Summary of Cohen’s kappa for inter-observer agreement, according to various scenarios

Scenario	Number of radiographs	Cohen’s kappa	Level of agreement	95% confidence interval
Overall cohort	1373	0.505	Moderate	0.455–0.554
Interpretations categorized as “Antibiotics Indicated” Versus “Not Indicated”	1373	0.508	Moderate	0.459–0.557
Radiographs interpreted by board-certified pediatricians	305	0.512	Moderate	0.405–0.618
Radiographs interpreted by residents	1068	0.495	Moderate	0.436–0.553
Radiographs obtained for patients with fever or dyspnea, to rule out bacterial pneumonia	1194	0.440	Moderate	0.411–0.470
Radiographs performed before midnight	1041	0.544	Moderate	0.489–0.600
Radiographs [performed after midnight	332	0.391	Fair	0.282–0.500

The expert panel concluded that for 1014 CRs (73.9% of the total), there was a “clinically relevant agreement” between the interpretations of the radiologists and the ED pediatricians. For 260 CRs (18.9%) there was “no-agreement,” and for 99 (7.2%) CRs, the experts agreed unanimously to categorize as “partial agreement.”

### Antibiotic prescriptions and other interventions

**Fig. 2:**
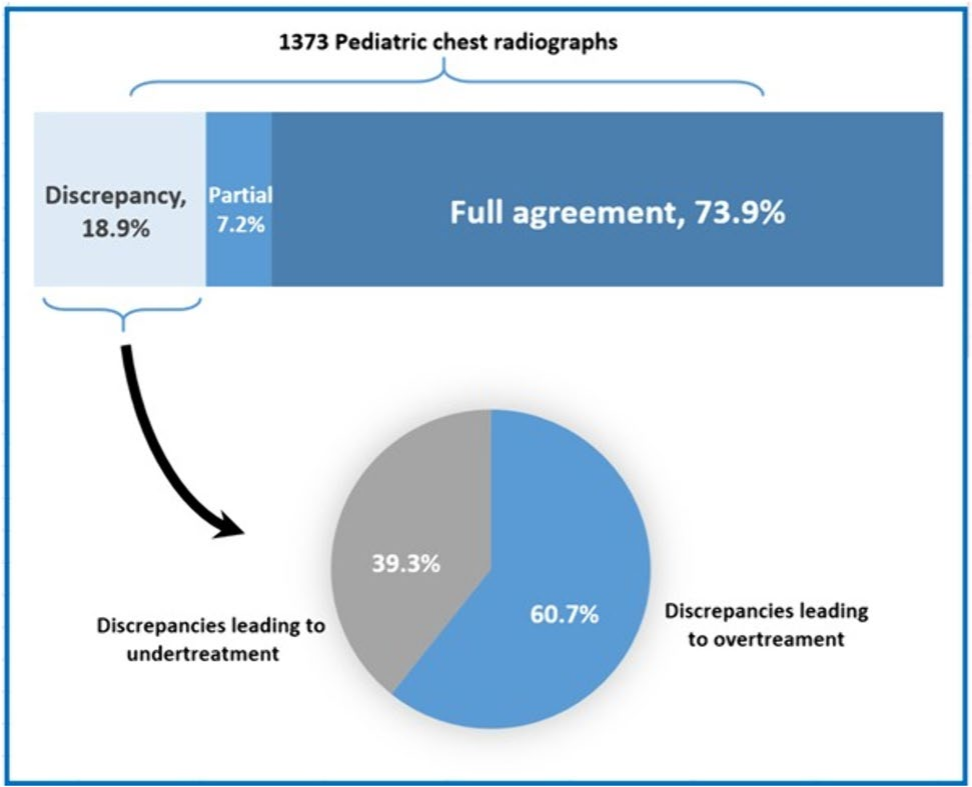
Clinically relevant agreement levels of chest radiographs according to the expert panel. Note: The pie chart summarizes only discrepancies related to antibiotic indications

In practice, antibiotic treatment was administered or recommended for 618 (45.0%) patients (due to numerous indications including bacterial pneumonia, suspected occult bacteremia, acute otitis media, and urinary tract infection). The expert panel concluded that in 122 CRs (8.9% of the whole cohort; 60.7% of the discrepancies related to antibiotic need), the prescription of the antibiotics was based on misinterpretation of the radiograph and therefore not justified. In contrast, among patients who were not recommended to take antibiotics, 79 (5.8% of the whole cohort; 39.3% of the discrepancies related to antibiotic need) were actually indicated to be treated with antibiotics according to the radiologists interpretations (Fig. 2).

Other interventions were required by the radiologists’ interpretation of 53 CRs (CT chest for clarifying inconclusive findings, pulmonologist referral for the suspicion of chronic lung disease, repeating CR in the future, etc.). Only for 23 CRs (43.3%) was the intervention conducted as recommended. It is important to note that the other patients and families were contacted by the study researchers and directed regarding required action.

### Risk factors for disagreement

To identify risk factors associated with diagnostic disagreement, the cohort was divided into two groups according to the expert panel attribution: “clinically relevant agreement” versus “disagreement.” The groups were compared according to parameters that could have been identified prior to the establishment of the final diagnosis (Table 3). Age, sex, and any medical background were similar between the groups.

**Table 3:** Demographic, clinical and laboratory parameters of the study population, according to clinically-relevant discrepancies (agreement and disagreement) in chest radiograph interpretation^a^

**Characteristic**	**Agreement** [*n* = 1014]	**Disagreement** [*n* = 260]	***p* value**^b,c^
**Epidemiological parameters**			
Age (years), median (IQR)	2.2 (1.1–5.4)	1.8 (1.1–4.2)	0.2
Sex, female	416 (41.0)	121 (46.5)	0.1
Medical background, any	271 (26.8)	77 (29.6)	0.4
Asthma or wheezing	75 (7.4)	23 (8.8)	0.4
Cardiologic background	39 (3.8)	12 (4.6)	0.6
Congenital/acquired immunodeficiency	53 (5.2)	9 (3.5)	0.2
Neurologic background^d^	27 (2.7)	13 (5.0)	0.054
Immunosuppressive treatment	28 (2.8)	2 (0.8)	0.058
Vaccination status (fully vaccinated)	672 (87.8)	176 (89.3)	0.6
Previous hospitalization^e^	99 (10.6)	27 (11.4)	0.7
Previous surgery	42 (4.4)	13 (5.3)	0.4953
Season, October—March	656 (64.7)	164 (63.1)	0.6794
Arrival to the ED by ambulance	74 (7.3)	20 (7.8)	0.8
**Clinical parameters**			
Antibiotics treatment before admission	160 (15.7)	37 (14)	0.5
Any respiratory treatment before admission	136 (13.6)	35 (13.7)	> 0.9
Disease length, median (IQR)	2 (1–5)	2.5 (1–5)	> 0.9
**Signs and symptoms**			
Fever ≥ 38°C	773 (76.5)	227 (87.3)	** < 0.001**
Chills	52 (5.8)	12 (5.2)	0.7
Cough, without dyspnea	458 (45.3)	129 (50.2)	0.2
Dyspnea	137 (14.2)	51 (20.0)	**0.011**
Any respiratory symptoms	682 (67.5)	197 (76.6)	**0.005**
Other, non-respiratory symptoms	504 (50.0)	106 (40.9)	**0.010**
Ill Appearance in physical exam	67 (6.8)	18 (7.1)	0.8
Abnormal respiratory findings in physical exam	395 (40.7)	142 (56.2)	** < 0.001**
Desaturation	61 (8.5)	24 (13.0)	0.2
CRP, mg/dL, median (IQR)	3.8 (1.2–7.9)	3.2 (1.3–7)	0.5
Total white blood cells, 10^3^ cells/µL, median (IQR)	13.6 (7.7–14.7)	13.9 (11.6–15.5)	0.4
**Treatments**			
Inhalation therapy	116 (11.4)	45 (17.3)	**0.011**
Fluid therapy	181 (17.8)	51 (19.6)	0.5
Antibiotics recommended/administrated	397 (39.7)	154 (59.4)	< 0.001
Hospital admission	206 (20.3)	56 (21.5)	0.7
**Chest radiograph parameters**			
Post-midnight acquisition	229 (22.6)	79 (30.4)	**0.009**
Weekends	314 (30.1)	80 (30.8)	> 0.9
Indication for the chest radiograph			**0.0075**
Respiratory symptoms	444 (43.8)	138 (53.1)	
Investigation of fever (patients without respiratory signs)	415 (40.9)	105 (40.4)	
Foreign body suspicion	44 (4.3)	8 (3.1)	
Chest pain/pressure	61 (6.1)	4 (1.5)	
Other	45 (4.4)	5 (1.9)	

The data are presented as *n* (%) unless stated otherwise

*IQR* interquartile range, *ED* emergency department, *CRP* C-reactive protein

^a^Based on expert panel review. Cases unanimously classified as “partial agreement” by the expert panel (*n* = 99) were omitted

^b^Wilcoxon rank sum test; Pearson’s chi-squared test; Fisher’s exact test

^c^*p* values < 0.05 are indicated in bold font, denoting statistical significance

^d^Neurologic background included cerebral palsy, autism spectrum disorder, epileptic disorder, ventriculoperitoneal (VP) shunt, muscle disorders, or bedridden patients due to severe developmental delay

^e^Within the last 3 months

The groups did not differ in disease duration or prior ambulatory respiratory treatment (inhalations, steroids, etc.). The “disagreement” group included higher proportions of children with fever [227 (87.3%) versus 773 (76.5%), *p* < 0.001] and with dyspnea [51 (20.0%) versus 137 (14.2%), *p* = 0.011]. The proportion that had cough without dyspnea was similar between the groups [129 (50.2%) versus 458 (45.3%), *p* = 0.2]. By contrast, non-respiratory symptoms were less common among the “disagreement” group [106 (40.9%) versus 504 (50.0%), *p* = 0.010]. For the “disagreement” compared to the “agreement” group, higher proportions of CRs were obtained after midnight [79 (30.4%) versus 229 (22.6%), *p* = 0.009] and were indicated due to respiratory symptoms [138 (53.1%) versus 444 (43.8%), *p* = 0.0075].

**Fig. 3:**
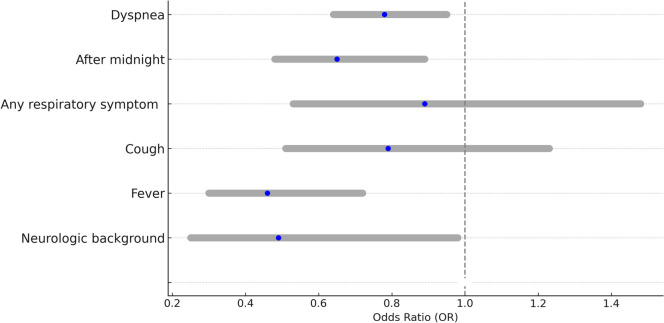
Variables associated with interpretation discrepancies in pediatric chest radiographs at the emergency department: binary logistic regression results (odds ratios with 95% confidence intervals); CR: chest radiograph

In a multivariable binary logistic regression (Fig. 3), the following parameters were found to be significantly associated with “disagreement”: neurological background (*p* = 0.046), fever (*p* = 0.001), CRs performed after midnight (*p* = 0.007), and dyspnea (*p* = 0.014). The other parameters examined did not achieve statistical significance.

## Discussion

The current study found moderate agreement (*k* = 0.505) between pediatricians at the ED and board-certified radiologists in interpreting pediatric CR. For 18.9% of the CRs, there was clinically relevant disagreement between interpreters. Antibiotic over-treatment was more common than under-treatment. Major clinical parameters that were associated with interpretation discrepancy were as follows: fever ≥ 38℃, prior neurological condition, dyspnea, and the interpretation occurring during the night shift.

CR is one of the most frequently performed imaging tests, being easy to perform, low in cost and readily available even in low-resource countries. CR provides important information regarding pediatric illness. However, their interpretation can be challenging, and significant variability and discrepancy in findings may lead to unnecessary medication or incorrect management. Fair to moderate levels of agreement, in the range of 0.2–0.68, have been reported [7, 8, 18–20]. Unlike most previous studies, the current study explored real-life clinical cases with “bedside” interpretation. Before conducting the study, we hypothesized that in these settings the level of agreement would be higher, as the patients’ clinical conditions could provide useful clues to interpret the CRs even for less experienced physicians. Our results are consistent with those reported by Soudack et al., who found discordant interpretations in 28% of patients in the ED.^5^ The upshot is that CR interpretation in real-life clinical situations should be taken with caution, and interpretation by a radiologist may aid in reaching a proper diagnosis.

A strength of the study is the unique approach, which focused on clinical, and not only radiological parameters, when identifying risk factors for disagreement. Clinical application of the model may help providers identify CRs that should be given high priority for obtaining interpretation by an experienced radiologist before reaching final clinical decisions. Additionally, these results can guide radiologists in setting priorities for interpretations, and potentially save economic resources while optimizing care for patients. [21].

Importantly, there is an inherent problem in identifying a “gold standard” in this field, as reaching an absolute diagnosis may require a complex investigation (such as a chest CT scan and bronchoalveolar lavage) which is not feasible (or recommended) for every patient requiring a chest X-ray. Under these conditions, an interpretation by a board-certified radiologist is considered the best *achievable* gold standard [15]. Indeed, some studies reported good inter-observer agreement among radiologists [22, 23]. However, clinical decisions result from a multitude of factors, of which imaging is just one. Therefore, the gold standard should be approached with caution, and measures like Cohen’s kappa, which examine agreement without bias towards a specific interpreter, should be preferred [14]. Accordingly, caution should be exercised regarding data related to the overuse/underuse of antibiotics, as antibiotic prescribing may reflect more than just a CR result. Nonetheless, this information is important for evaluating the clinical significance of the major findings and was reported in other studies as well. [5, 18, 24]. Notably, most other findings in this study (including the clinical score) are based on agreement between interpreters, without indicating the “correct” interpreter.

We concluded that the most significant parameters for incorrect interpretation were as follows: fever ≥ 38℃, a background neurological condition, dyspnea, and the interpretation occurring during the night shift. We assume that patients with background neurological conditions show higher tendency for scoliosis and chronic lung diseases. These obscure physicians’ interpretations of the CRs, with many CRs taken bedside, leading to technical issues and artifacts. [25]. Regarding night shifts, this is known to prompt medical errors, whether due to the predominance of trainees during these hours, with the lack of experienced physicians to consult with; or due to physician fatigue. [26–28]. Interestingly, unlike others [7, 8], we did not find a statistically significant difference between the level of agreement within the group of board-certified pediatricians compared to the level of agreement within the group of resident pediatricians. We speculate that this may be due to the very high proportion of trainees in the general cohort (which is apparently related to the pre-selected times that are considered off-hours).

The higher risk of disagreement in the context of fever ≥ 38℃ and dyspnea could be related to the higher probability of lower airway involvement, thus complicating the interpretation.^14^ Alternatively, familiarity with the patient’s clinical symptoms may subconsciously affect the interpretation of CR. Either way, extra caution must be taken when interpreting CR in these contexts.

In many hospitals and imaging centers, conventional films were gradually replaced by Picture Archiving and Communication Systems (PACS). These changed have economic benefits [29] but their influence on the accuracy of interpretation is less clear. Nevertheless, some studies reported that radiograph interpretations with digital studies remain as accurate as assessments performed using conventional radiographs, [30, 31] or even that sensitivity was improved after introduction of PACS [32]. Although the current study was conducted with digital high-resolution images, it is difficult to compare the results to other studies in the field, as not all the authors designated the specific technology they used. Of note, the future introduction of artificial intelligence to EDs may potentially increase the accuracy of CR interpretations. [33]. Studies are needed to estimate this improvement.

### Limitations

The retrospective design is a limitation of this work, which precludes access to the complete clinical picture of every patient and the establishment of the final role of CR in patient management. To overcome this, an expert panel reviewed every patient’s medical record, in an effort to mitigate bias. As discussed above, there is no absolute usable gold-standard for CR; thus, most of the results in this study were based on level of agreement rather than on “incorrect interpretations.” In addition, the study was conducted in a tertiary pediatric hospital with a significant rate of patient complexity, together with a high patient volume. This sometimes necessitated rapid decision-making, and the results should be considered in the relevant context. As several parameters (not only CR interpretation) are considered in clinical decisions, the fundamental necessity of providing a radiologist’s interpretation for CRs cannot be concluded from this study, but rather only the proportion of CRs with discrepancy in interpretation and their characteristics. Finally, the WHO assessment method of CR mentioned above is not relevant to some of the less prevalent diagnoses (including foreign body in airways and pneumothorax).

## Conclusions

In conclusion, we report a moderate level of agreement between pediatric providers and board-certified radiologists in interpreting CR in ED settings. Disputed CRs resulted in more antibiotics overtreatment than undertreatment. Clinical risk factors for disagreement are presented, with the aim of identifying CRs at high risk for disagreement in interpretation. Implementing these results at the pediatric ED can facilitate the utilization of radiologists’ expertise, save time and resources, and potentially improve patient care.

## Supplementary Information

Below is the link to the electronic supplementary material.

Supplementary file1 Regular working hours (green) versus other hours, including night shifts and weekends (pink) in our center (JPG 52 KB)

## Data Availability

The data that support the findings of this study are available from the corresponding author, [YL], upon reasonable request.

## Abbreviations

CR: Chest radiograph
CRP: C-reactive protein
ED: Emergency department

## References

[1] Neuman MI, Graham D, Bachur R (2011) Variation in the use of chest radiography for pneumonia in pediatric emergency departments. Pediatr Emerg Care 27(7):606–610 21712748 10.1097/PEC.0b013e3182225578

[2] Geanacopoulos AT, Porter JJ, Monuteaux MC, Lipsett SC, Neuman MI (2020) Trends in chest radiographs for pneumonia in emergency departments. Pediatrics 145(3):e20192816. 10.1542/peds.2019-2816 32079719 10.1542/peds.2019-2816

[3] De Lange C (2011) Radiology in paediatric non-traumatic thoracic emergencies. Insights Imaging 2(5):585–598. 10.1007/s13244-011-0113-4 22347978 10.1007/s13244-011-0113-4 PMC3259402

[4] Bradley JS , Byington CL , Shah SS et al (2011) Pediatric Infectious Diseases Society; Infectious Diseases Society of America . The management of community-acquired pneumonia in infants and children older than 3 months of age: clinical practice guidelines by the Pediatric Infectious Diseases Society and the Infectious Diseases Society of America. Clin Infect Dis 53(7):e25–e76 10.1093/cid/cir531 PMC7107838 21880587

[5] Soudack M, Raviv-Zilka L, Ben-shlush A, Jacobson JM, Benacon M, Augarten A (2012) Who should be reading chest radiographs in the pediatric emergency department? Pediatr emer care 28:1052–1054 10.1097/PEC.0b013e31826caf3f 23023476

[6] Menashe SJ, Iyer RS, Parisi MT, Otto RK, Stanescu AL (2016) Pediatric chest radiographs: common and less common errors. AJR Am J Roentgenol 207(4):903–911. 10.2214/AJR.16.16449 27490235 10.2214/AJR.16.16449

[7] Voigt GM, Thiele D, Wetzke M, Weidemann J, Parpatt P-M, Welte T, Seidenberg J, Vogelberg C, Koster H, Rhode GGU, Hartel C, Hensen G, Kopp MV (2021) interobserver agreement in interpretation of chest radiographs for pediatric community aquired pneumonia: findings of the pedACPNETZ-cohort. Pediatr Pulmonol 56:2676–2685 34076967 10.1002/ppul.25528

[8] Johnson J, Kline JA (2010) intraobserver and interobserver agreement of the interpretation of pediatric chest radiograph. Emerg radiol 17:285–290 20091078 10.1007/s10140-009-0854-2

[9] Mehrotra P, Bosemani V, Cox J (2009) Do radiologists still need to report chest x rays? Postgrad Med J 85(1005):339–341. 10.1136/pgmj.2007.066712 19581241 10.1136/pgmj.2007.066712

[10] Eng J, Mysko WK, Weller GE, Renard R, Gitlin JN, Bluemke DA, Magid D, Kelen GD, Scott WW Jr (2000) Interpretation of Emergency Department radiographs: a comparison of emergency medicine physicians with radiologists, residents with faculty, and film with digital display. AJR Am J Roentgenol 175(5):1233–1238. 10.2214/ajr.175.5.1751233 11044013 10.2214/ajr.175.5.1751233

[11] Levinsky Y, Mimouni FB, Fisher D, Ehrlichman M (2013) Chest radiography of acute paediatric lower respiratory infections: experience versus interobserver variation. Acta pediatrica 102:e310–e314 10.1111/apa.12249 23565882

[12] Nesterova GV, Leftridge CA Jr, Natarajan AR, Appel HJ, Bautista MV, Hauser GJ (2010) Discordance in interpretation of chest radiographs between pediatric intensivists and a radiologist: impact on patient management. J Crit Care 25(2):179–183. 10.1016/j.jcrc.2009.05.016. 19682850 10.1016/j.jcrc.2009.05.016

[13] Rowe S, O’Riordan P, Woznitza N (2019) Greater than the sum of the parts: impact of radiographer clinical image interpretation. J Med Radiat Sci 66:149–151 31449741 10.1002/jmrs.342 PMC6745340

[14] McHugh ML (2012) Interrater reliability: the kappa statistic. Biochem Med (Zagreb) 22(3):276–282 23092060 PMC3900052

[15] Cherian T, Mulholland EK, Carlin JB, Ostensen H, Amin R, de Campo M (2005May) Standardized interpretation of paediatric chest radiographs for the diagnosis of pneumonia in epidemiological studies. Bull World Health Organ 83(5):353–359 15976876 PMC2626240

[16] Buenger RE (1988) Five thousand acute care/emergency department chest radiographs: comparison of requisitions with radiographic findings. J Emerg Med 6(3):197–202. 10.1016/0736-4679(88)90326-5 3171120 10.1016/0736-4679(88)90326-5

[17] Shoukri MM, Asyali MH, Donner A (2004) Sample size requirements for the design of reliability study: review and new results. Stat Methods Med Res 13:1–2114746437

[18] Bada C, Carreazo NY, Chalco JP, Huicho L (2007) Inter-observer agreement in interpreting chest X-rays on children with acute lower respiratory tract infections and concurrent wheezing. Sao Paulo Med J 125(3):150–154. 10.1590/s1516-31802007000300005 17923939 10.1590/S1516-31802007000300005 PMC11020574

[19] Williams GJ, Macaskill P, Kerr M, Fitzgerald DA, Isaacs D, Codarini M, McCaskill M, Prelog K, Craig JC (2013) Variability and accuracy in interpretation of consolidation on chest radiography for diagnosing pneumonia in children under 5 years of age. Pediatr Pulmonol 48(12):1195–1200. 10.1002/ppul.22806 23997040 10.1002/ppul.22806

[20] Robinson PJ, Wilson D, Coral A, Murphy A, Verow P (1999) Variation between experienced observers in the interpretation of accident and emergency radiographs. Br J Radiol 72(856):323–330. 10.1259/bjr.72.856.10474490 10474490 10.1259/bjr.72.856.10474490

[21] Sailer AM, van Zwam WH, Wildberger JE, Grutters JP (2015) Cost-effectiveness modelling in diagnostic imaging: a stepwise approach. Eur Radiol 25(12):3629–3637 26003789 10.1007/s00330-015-3770-8 PMC4636534

[22] Klein EJ, Koenig M, Diekema DS, Winters W (1999) Discordant radiograph interpretation between emergency physicians and radiologists in a pediatric emergency department. Pediatr Emerg Care 15(4):245–248 10460076

[23] Davies HD, Wang EE, Manson D, Babyn P, Shuckett B (1996) Reliability of the chest radiograph in the diagnosis of lower respiratory infections in young children. Pediatr Infect Dis J 15(7):600–604. 10.1097/00006454-199607000-00008 8823854 10.1097/00006454-199607000-00008

[24] Xavier-Souza G, Vilas-Boas AL, Fontoura MS, Araújo-Neto CA, Andrade SC, Cardoso MR, Nascimento-Carvalho CM, PNEUMOPAC-Efficacy Study Group (2013) May The inter-observer variation of chest radiograph reading in acute lower respiratory tract infection among children. Pediatr Pulmonol 48(5):464–9. 10.1002/ppul.22644 10.1002/ppul.22644 22888091

[25] Eisenhuber E, Schaefer-Prokop CM, Prosch H, Schima W (2012) Bedside chest radiography. Respir Care 57(3):427–443. 10.4187/respcare.01712 22391269 10.4187/respcare.01712

[26] Klinefelter Z, Hirsh EL, Britt TW, George CL, Sulzbach M, Fowler LA (2023) Shift happens: emergency physician perspectives on fatigue and shift work. Clocks Sleep 5(2):234–248 37092431 10.3390/clockssleep5020019 PMC10123702

[27] Williamson A, Lombardi DA, Folkard S, Stutts J, Courtney TK, Connor JL (2011) The link between fatigue and safety. Accid Anal Prev 43(2):498–515. 10.1016/j.aap.2009.11.011 21130213 10.1016/j.aap.2009.11.011

[28] Gates M, Wingert A, Featherstone R, Samuels C, Simon C, Dyson MP (2018) Impact of fatigue and insufficient sleep on physician and patient outcomes: a systematic review. BMJ Open 8(9):e021967 30244211 10.1136/bmjopen-2018-021967 PMC6157562

[29] Kim SA, Park WS, Chun TJ, Mo NC (2002Dec) Association of the implementation of PACS with hospital revenue. J Digit Imaging 15(4):247–253. 10.1007/s10278-002-0025-4 12488966 10.1007/s10278-002-0025-4 PMC3611615

[30] Hurlen P, Borthne A, Dahl FA, Ostbye T, Gulbrandsen P (2012) Does PACS improve diagnostic accuracy in chest radiograph interpretations in clinical practice? Eur J Radiol 81(1):173–177. 10.1016/j.ejrad.2010.08.043 20888718 10.1016/j.ejrad.2010.08.043

[31] Frank MS, Jost RG, Molina PL, Anderson DJ, Solomon SL, Whitman RA, Moore SM (1993) High-resolution computer display of portable, digital, chest radiographs of adults: suitability for primary interpretation. AJR Am J Roentgenol 160(3):473–477. 10.2214/ajr.160.3.8430538 8430538 10.2214/ajr.160.3.8430538

[32] Gouin S, Patel H, Bergeron S, Amre D, Guérin R (2006) The effect of Picture Archiving and Communications Systems on the accuracy of diagnostic interpretation of pediatric emergency physicians. Acad Emerg Med 13(2):186–190. 10.1197/j.aem.2005.08.006 16436791 10.1197/j.aem.2005.08.006

[33] Bennani S, Regnard NE, Ventre J, Lassalle L, Nguyen T, Ducarouge A, Dargent L, Guillo E, Gouhier E, Zaimi SH, Canniff E, Malandrin C, Khafagy P, Koulakian H, Revel MP, Chassagnon G (2023) Using AI to improve radiologist performance in detection of abnormalities on chest radiographs. Radiology 309(3):e230860. 10.1148/radiol.230860 38085079 10.1148/radiol.230860

