# Supplementary figures and images for: Pediatric chest radiograph interpretation in a real-life setting
Source: Eur J Pediatr. 2024 Aug 12;183(10):4435–44. doi: 10.1007/s00431-024-05717-x (PMC11413054; doi:10.1007/s00431-024-05717-x)

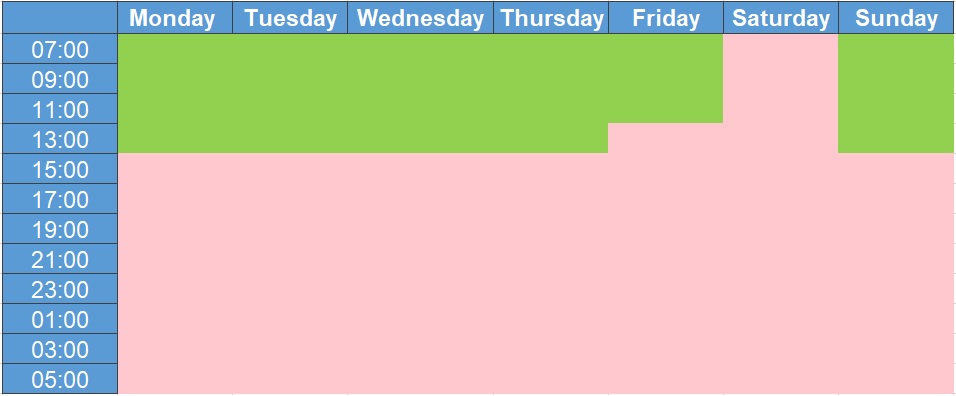

Supplement: Supplementary file 1 — Supplementary file1 Regular working hours (green) versus other hours, including night shifts and weekends (pink) in our center (JPG 52 KB) [file 431_2024_5717_MOESM1_ESM.jpg]
